# *In vitro* Evidence That Combination Therapy With CD16-Bearing NK-92 Cells and FDA-Approved Alefacept Can Selectively Target the Latent HIV Reservoir in CD4+ CD2hi Memory T Cells

**DOI:** 10.3389/fimmu.2018.02552

**Published:** 2018-11-05

**Authors:** Amanda G. Tomalka, Ivelisse Resto-Garay, Kerry S. Campbell, Daniel L. Popkin

**Affiliations:** ^1^Department of Dermatology, Case Western Reserve University School of Medicine, Cleveland, OH, United States; ^2^Blood Cell Development and Function Program, Fox Chase Cancer Center, Institute for Cancer Research, Philadelphia, PA, United States

**Keywords:** HIV, ADCC, alefacept, NK, NK92, FDA, CD2, CD16

## Abstract

Elimination of the latent HIV reservoir remains the biggest hurdle to achieve HIV cure. In order to specifically eliminate HIV infected cells they must be distinguishable from uninfected cells. CD2 was recently identified as a potential marker enriched in the HIV-1 reservoir on CD4+ T cells, the largest, longest-lived and best-characterized constituent of the HIV reservoir. We previously proposed to repurpose FDA-approved alefacept, a humanized α-CD2 fusion protein, to reduce the HIV reservoir in CD2hi CD4+ memory T cells. Here, we show the first evidence that alefacept can specifically target and reduce CD2hi HIV infected cells *in vitro*. We explore a variety of natural killer (NK) cells as mediators of antibody-dependent cell-mediated cytotoxicity (ADCC) including primary NK cells, expanded NK cells as well as the CD16 transduced NK-92 cell line which is currently under study in clinical trials as a treatment for cancer. We demonstrate that CD16.NK-92 has a natural preference to kill CD2hi CD45RA– memory T cells, specifically CD45RA– CD27+ central memory/transitional memory (T_CM/TM_) subset in both healthy and HIV^+^ patient samples as well as to reduce HIV DNA from HIV^+^ samples from donors well controlled on antiretroviral therapy. Lastly, alefacept can combine with CD16.NK-92 to decrease HIV DNA in some patient samples and thus may yield value as part of a strategy toward sustained HIV remission.

## Introduction

HIV infection remains one of the greatest global health challenges of our time. Despite heroic advances in antiviral therapy (ART), which utilizes a combination of antiretroviral drugs to target multiple steps of the HIV life cycle and suppresses plasma viremia below the limit of detection of clinical assays, a pool of latent HIV infected cells remain (1, 2). These latently infected HIV^+^ cells, largely the long lived CD4+ resting memory T cells, remain in a quiescent state and harbor replication competent virus which contributes to viral rebound generally within 2 weeks if antiretroviral therapy is ceased or interrupted (3–9). To date, there has been only one case of functional HIV cure, the “Berlin patient” who received two hematopoietic stem cell transplants as treatment for leukemia and subsequent relapse. The donor grafted cells contained the CCR5Δ32 mutation, rendering them resistant to HIV infection as they repopulated the patient and were preserved despite an interruption in ART (10). It has been estimated that the “Berlin patient” experienced more than a 3.5 log reduction in HIV reservoir due to the transplant and has yet to experience viral rebound (11, 12). However effective in the “Berlin patient,” hematopoietic stem cell transplantation is not a viable cure option for the millions of people infected with HIV worldwide and the 2 million new infections annually. Thus, a readily deployable cure or any scalable strategy to obtain sustained virologic remission for HIV remains elusive.

As HIV cure remains elusive, so does the search for true markers of the HIV reservoir upon which cure strategies can be developed and directed. CD32 had been recently proposed as a marker of the CD4+ T cell HIV reservoir, however these findings have not been reproducible. Instead, it was observed that CD32 may rather mark a small subset of transcriptionally active HIV^+^ cells and not the latent reservoir in resting memory cells (13–20). In an unbiased microarray analysis, CD2 has been identified and validated as the best marker for cells bearing both HIV proviral DNA as well as inducible HIV RNA from 6 donors with well controlled viral loads on antiretroviral therapy (21).

Although CD2 is present on all T lymphocytes, memory T cells bear the highest density of CD2, an adhesion molecule and co-receptor (22, 23). Alefacept, FDA-approved as the first biologic for the treatment of psoriasis in 2002, is a fusion protein of the natural ligand of CD2, LFA3 to human IgG1. Alefacept exhibits a dual mechanism of action first by blocking the natural interaction of CD2 with its ligand LFA3 (CD58) on an antigen presenting cell which inhibits T cell activation. Additionally, the IgG1 backbone of alefacept binds to the FcγRIII receptor, CD16, on natural killer (NK) cells to initiate antibody-dependent cell-mediated cytotoxicity (ADCC) of CD2hi pathologic memory T cells by inducing granzyme mediated apoptosis (24–30). This dual mechanism of action both prevents the activation of memory T cells as well as eliminates them. In clinical trials this effect was observed only for CD4+ and CD8+ CD45RO+ memory T cells in a dose dependent manner. Alefacept had no effect on naïve CD4+ or CD8+ CD45RA+ T cells, CD19+ B cells, or CD16+ CD56+ NK cells *in vivo* (31). At 12 weeks post therapy, most patients have returned their total lymphocyte counts to normal levels (32). Alefacept has an excellent safety profile. In clinical trials, no opportunistic infections or viral herpes reactivation were reported and there was no increased incidence of malignancy. Overall, alefacept is well tolerated and effective in depleting CD2hi memory T cells *in vivo* and improving psoriasis outcomes (32–37).

Ultimately, we hypothesize that eliminating CD2hi CD4+ memory T cells may contribute to HIV reservoir reduction in some individuals. Importantly, HIV infected cells are not the only cells that express CD2. CD2 is expressed on CD4+ and CD8+ T cells as well as NK cells. Thus, we sought to determine if alefacept may be repurposed to enrich for killing of T cells bearing HIV *in vitro* vs. HIV^−^ T cells and NK cells in defined *in vitro* culture models. Here we have investigated *in vitro* interventions combining alefacept with NK cells (the most prominent effector of ADCC) to selectively decrease HIV latently infected CD4+ T cells from peripheral blood. These data support the potential of repurposing FDA-approved alefacept to safely and effectively reduce the CD2hi HIV reservoir that exists in CD4+ memory T cells, leading to long term control of the virus. However, we acknowledge that HIV^+^ cells will not be specifically targeted and that CD2+ bystander cells may also be eliminated. Our strategy may better be described as reducing the number of CD2+ cells and as a result of that HIV^+^ cells are also eliminated. Overall, we seek to find a readily implementable strategy that can be tolerated in our patients to decrease the HIV reservoir. Given the extremely difficult task at hand, we posit that our strategy may provide some added benefit to other approaches as it is not mutually exclusive with “kick and kill” and other related approaches and could be tolerated similarly well as in psoriasis patients who received this drug in 2002 and thereafter. To begin addressing this hypothesis, we explored a variety of NK cells as mediators of ADCC to target the HIV reservoir and show that CD16.NK-92 has a natural preference for CD45RA– memory T cells without the need for viral reactivation, avoiding possible pitfalls of a “kick and kill” approach and at minimum providing a complementing “kill” strategy that does not require potentially toxic “kick” drugs that do not provide 100% latency reversal (2). We utilized the most sensitive and accurate measure of cytotoxicity enumeration with low effector:target cell (E:T) ratios, absolute count flow cytometry, to account for every cell in the ADCC co-culture to yield highly precise and robust measures of specific cytotoxicity with alefacept. Additionally, absolute count flow cytometry enumeration of surviving target cells yielded a lower baseline lysis and higher maximum lysis than other techniques compared side-by-side at low E:T ratios (38). This results in more sensitive detection with a larger dynamic range for the assays we performed. Physiologically, we reasoned that low E:T ratios are relevant.

## Materials and methods

### Cells and cell culture

Healthy donor PBMCs were obtained from American Red Cross (Cleveland, OH) Leukocyte reduction filters (LRFs) as discarded medical waste and PBMCs isolated on a density gradient of Lymphoprep (STEMCELL Technologies) and immediately cryopreserved in 90% FBS (Seradigm) and 10% DMSO (Sigma) at 5 × 10^6^ cells/mL.

HIV^+^ donor PBMCs were obtained from CFAR Clinical Core (Cleveland, OH) leukaphereses from ART treated patients with at least two undetectable viral loads over the year prior to donating. PBMCs were isolated and cryopreserved as described above.

Primary NK cells from healthy donors were enriched from cryopreserved PBMCs using EasySep Human NK Cell Enrichment Kit (STEMCELL Technologies) and rested overnight at 37°C and 5% CO_2_ in RPMI 1640 (LRI Central Cell Services) supplemented with 10% FBS (Seradigm), 2 mM L-glutamine, 25 mM HEPES, 100 IU/mL penicillin, 100 μg/mL streptomycin (all GenClone), hereafter referred to as complete RPMI, and 20 IU/mL recombinant human IL-2 (Peprotech).

Jurkat cell lines E6.1 (ATCC® TIB-152^TM^) and 3C9 (HIV^+^) (39) were maintained in complete RPMI. K562 Cl9 mIL21 feeder cells (40) were also maintained in complete RPMI, γ-irradiated with 50 Gy and cryopreserved in 90% FBS and 10% DMSO at 3 × 10^6^ cells/mL until required for NK cell expansion.

Primary CD4+ T cells (healthy donor and ART treated/controlled viral load HIV^+^) were enriched from cryopreserved PBMCs with EasySep Human CD4+ T cell Enrichment Kit (STEMCELL Technologies) and rested overnight in complete RPMI and 20 IU/mL recombinant human IL-2 (Peprotech).

CD16.NK-92 was maintained in complete NK-92 media: α-MEM with deoxyribonucleosides and ribonucleosides (LRI Central Cell Services) supplemented with 10% FBS (Seradigm), 10% horse serum, 0.1 mM β-mercaptoethanol (both Gibco), 1X non-essential amino acids, 1 mM sodium pyruvate, 2 mM L-glutamine, 100 IU/mL penicillin, 100 μg/mL streptomycin (all GenClone), 0.2 mM myo-Inositol and 0.0025 mM folic acid (both Sigma) and supplemented with 10% culture supernatant of J558L cells transfected with the human IL-2 gene (41, 42).

### Flow cytometry

Flow cytometry was performed on a MacsQuant 10 Analyzer (Miltenyi Biotec) and data was analyzed using FlowJo (Tree Star).

### Cytotoxicity assays with absolute count flow cytometry

Effector and target cells were mixed and co-cultured for 19 h at 37°C and 5% CO_2_ in complete RPMI media unless otherwise indicated with 50 IU/mL recombinant human IL-2 with either 10 μg/mL alefacept (Biogen) or IgG1 kappa isolated from human myeloma plasma (Sigma) for 19 h unless otherwise indicated. Isotype control IgG1, kappa was chosen as it has been shown to bind to CD16, the Fc receptor, and lead to ADCC (43–45). Additionally, it has been demonstrated that to observe the effects of alefacept *in vivo*, binding of alefacept IgG1 backbone to the Fc receptor is required (46). After co-culture, cells were stained with antibodies and viability dye with no wash. Cytotoxicity was enumerated by flow cytometry using absolute cell counts where an equal volume of stained co-culture was sampled from each experimental condition allowing the exact cell counts of each cell type in the culture The same number of cells were added to the experimental (effector and target cell co-culture) and control wells (effector or target cell type alone) such that when the experimental co-culture absolute cell number (actual cell number) is compared to control wells (expected cell number) the percent cytotoxicity can be calculated as described below. This allows for the enumeration of not only cells that are dead by viability dye but also cells that have been killed and are missing from the co-culture.

Primary NK cells from healthy donors and Jurkat mix cytotoxicity assay: 3C9 HIV^+^ latent Jurkat were either pre-stimulated with 1 ng/mL TNFα for 19 h to induce expression of HIV-GFP or left unstimulated. Prior to start of assay, TNFα was washed away and 3C9 was pre-labeled with 0.5 uM CellTracker Deep Red Dye and E6.1 HIV^−^ parental Jurkat was pre-labeled with 0.5 uM CellTrace CFSE Cell Proliferation Kit (both ThermoFisher Scientific), according to manufacturer instructions. Deep red labeled HIV^+^ 3C9 was diluted 1:100 into CFSE labeled HIV^−^ E6.1 to create a Jurkat Mix of target cells containing 1 × 10^4^ deep red stained 3C9 cells and 9.9 × 10^5^ CFSE stained E6.1 cells. The fluorescence emission from the two stained cell types allows for signal isolation as the HIV-GFP 3C9 cells are also emitting within the deep red cell tracker spectrum (APC-Cy7 channel) whose Excitation/Emission properties make it completely spectrally isolated from the CFSE/GFP channel (aka “FL1”). 1 × 10^5^ primary NK cells from healthy donors were added to the Jurkat mix to yield an effector:target cell ratio (E:T) of 0.1:1 and remain unlabeled.

Primary and expanded NK and CD4+ T cells cytotoxicity assay: 5 × 10^4^ healthy donor primary NK cells (day 0) or expanded NK cells (day 21) were used as effector cells at an E:T = 1:1 with either 5 × 10^4^ self (autologous), hereafter referred to as auto, CD4+ T cells or non-self (allogeneic), hereafter referred to as allo, CD4+ T cells as target cells.

CD16.NK-92 and CD4+ T cells cytotoxicity assay: 1 × 10^3^-1 × 10^5^ CD16.NK-92 and 1 × 10^5^ healthy donor CD4+ T cells were co-cultured in complete NK-92 media with E:T ratios ranging from 0.01:1 to 1:1. HIV^+^ co-cultures were extended for 3 days with a sample taken at 19 h for day 1 enumeration of cytotoxicity by flow cytometry with E:T ratio of 0.5:1 where CD16.NK92 effectors ranged from 7.5 × 10^5^ to 3.5 × 10^6^ and HIV^+^ CD4+ T cell targets ranged from 1.5 × 10^6^ to 7 × 10^6^ depending on cell numbers available. Where indicated, CD16.NK-92 was γ-irradiated with 10 Gy prior to start of assay.

### NK cell expansion

One day prior to addition of feeder cells, 50 Gy γ-irradiated cryopreserved K562 Cl9 mIL21 (40) were thawed and rested overnight in complete RPMI. Four primary NK cell healthy donors were expanded as previously described (47), with or without the addition of 10 μL (5 μg) BD FastImmune^TM^ anti-human CD2 (clone L303.1, BD Biosciences) at days 0, 7, and 14 of the 21 day expansion protocol. BD FastImmune^TM^ anti-human CD2 was only present during the expansion and cells were washed prior to phenotyping by surface stain at days 0, 7, 14, and 21.

### Flow cytometry antibodies and dyes

α-CD2-PerCP-Vio770 (clone LT2, Miltenyi Biotec), NFL1 viability dye (OncoImmunin), CD56-BV421 (clone HCD56, Biolegend), CD2-BV510 (clone RPA-2.10, Biolegend), CD4-PEVio770 (clone REA623, Miltenyi Biotec), CD45RA-APC (clone REA562, Miltenyi Biotec), CD3-APC/Cy7 (clone SK7, Biolegend), propidium iodide (Miltenyi Biotec), CCR7-BV421 (clone 3D12, BD Biosciences), CD27-PE (clone REA499, Miltenyi Biotec), CD16-APCVio770 (clone REA426, Miltenyi Biotec) and CD56-PEVio770 (clone REA196, Miltenyi Biotec).

### Equations and calculations

Percent cytotoxicity was calculated as previously described (38). Briefly, % cytotoxicity = (expected cell number) – (actual cell number) / (expected cell number) ^*^100, where the expected cell number is the absolute cell count from a control sample of target or effector cells alone and the actual cell number is the absolute cell count from an experimental sample.

### Quantitative PCR

Cell pellets were collected after 3 day co-culture with CD16.NK-92 and 10 μg/mL alefacept or IgG1 control antibody. Total DNA was isolated from cell pellets using TRIzol reagent (Invitrogen) in accordance with the manufacturer instructions. Quantitative PCR was performed in triplicate on each sample using TaqMan® Fast Advanced Master Mix Kit (ThermoFisher Scientific). Amplification reactions contained 0.2–1 μg of sample DNA (depending on DNA yield after DNA extraction of each sample) in a final volume of 40 μL. Sense primer SK462 (5′-AGT TGG AGG ACA TCA AGC AGC CAT GCA AAT-3′) and antisense primer SK431 (5′-TGC TAT GTC AGT TCC CCT TGG TTC TCT-3′) were used in conjunction with FAM-labeled probe SK102 (5′-AGA CCA TCA ATG AGG AAG CTG CAG AAT GGG AT-3′), each at a final concentration of 100 nM, to amplify the *gag* region of HIV. RPP30-specific sense and antisense primers (5′-GAT TTG GAC CTG CGA GCG-3′ and 5′-GCG GCT GTC TCC ACA AGT-3′, respectively) with VIC-labeled probe, each at a final concentration of 100 nM, were used to calculate the amount of CD4+ T cells in each sample using a StepOnePlus real-time PCR instrument (Applied Biosystems). After initial incubation of 95°C for 5 min, 45 cycles of amplification were carried out at 98°C for 10 s followed by 1 min at 60°C. The amount of *gag* DNA was initially plotted as HIV *gag* DNA copies per well and fitted to an ACH2 standard curve with serial dilutions of 1000-1 copy ACH2 per well in 10-fold increments in triplicate. All calibration points of the ACH2 standard curve contained a constant background of 0.3 μg uninfected cellular DNA. Additional cellular DNA up to 2 μg/well had negligible effect on sensitivity and specificity, consistent with previously reported findings with this assay (48). All experiments included a standard curve for every run and multiple wells containing the complete reaction cocktail with water spike-in negative controls. qPCR was deemed interpretable if control rows for said experiment including positive, negative and standard curve 10-fold dilutions were as expected. Experimental samples below 1 copy HIV *gag* DNA per well were deemed not determined (N.D.). Values above the limit of detection were then represented as HIV *gag* DNA copies/10^6^ CD4+ T cells. At least 600,000 cell equivalents were accessed for each sample, thus a conservative limit of detection of 2 copies/10^6^ cells was used and represented as a dotted line in all relevant figures.

### Statistical analyses

GraphPad Prism version 6.00 for Windows (GraphPad Software) was used for all statistical analyses and graphing. For CD2 MFI, fold expansion and percent positive, statistics are two-tailed paired parametric *t*-tests. For percent cytotoxicity between IgG1 vs. alefacept, absolute count and HIV copies/million CD4+ T cells statistics are one-tailed parametric paired *t-*tests as prior clinical data has shown reduction of CD2hi memory T cells with alefacept (33). For comparisons between cell type groups (i.e., % cytotoxicity of CD45RA+ vs. CD45RA– treated with IgG1), two-tailed parametric paired *t* tests were used as differences were unprecedented. Error bars represent SEM, ns *P* > 0.05, ^*^p ≤ 0.05, ^**^p < 0.01, ^***^p < 0.001, ^****^p < 0.0001.

## Results

### HIV infection and reactivation of virus increases CD2 MFI in jurkat model

**Figure 1:**
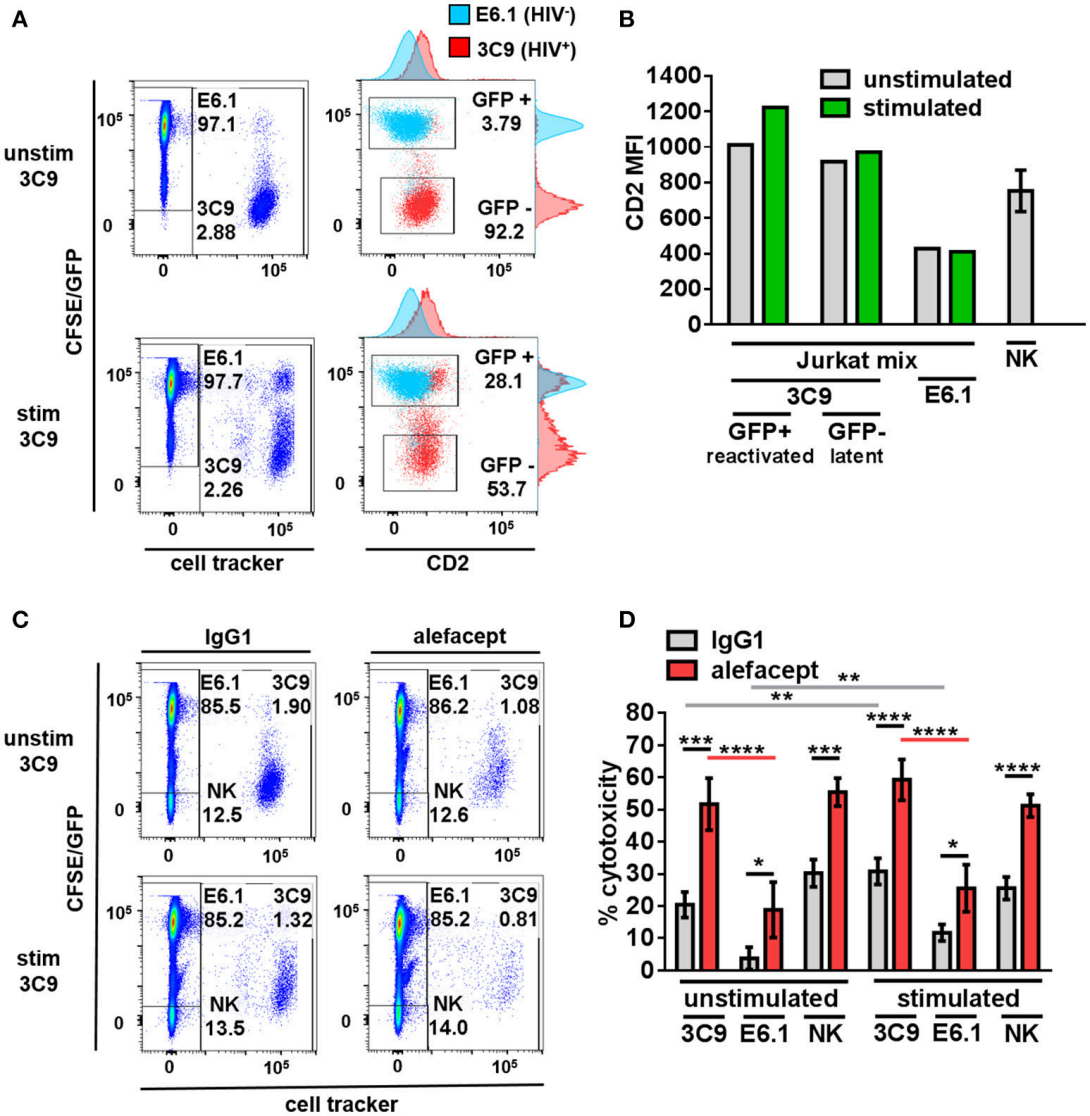
Primary NK cells selectively target HIV^+^ 3C9 Jurkat for killing by ADCC with alefacept more than HIV^−^ E6.1 in Jurkat mix. **(A)** Flow cytometry plots of Jurkat mix. Deep red cell tracker labeled HIV^+^ 3C9 was diluted 1:100 with CFSE labeled uninfected E6.1 parent Jurkat cell line. 3C9 Jurkat was either unstimulated or pre-stimulated with 1 ng/mL TNFα before mixing with E6.1 Jurkat to induce expression of GFP-HIV. **(B)** Experimental representative of quantification of CD2 MFI on Jurkat cells and *n* = 6 primary NK cell donors. **(C)** Flow cytometry plots of representative donor primary NK cells and Jurkat mix killing assay with 10 μg/mL IgG1 or alefacept. **(D)** Percent cytotoxicity of 3C9, E6.1 and primary NK cells with IgG1 control antibody or alefacept after co-culture as measured by absolute cell count flow cytometry with *n* = 6 primary NK cell donors with E:T = 0.1:1, after 19 h in co-culture Mean and SEM shown for 6 primary NK cell donors, *P ≤ 0.05, **P ≤ 0.01, ***P ≤ 0.001, ****P ≤ 0.0001. Statistical significance between IgG1 and alefacept conditions are indicated with black bars, statistical significance between cell types with IgG1 are indicated with gray bars and statistical significance between cell types with alefacept are indicated with red bars. Absolute CD2 MFI and relative MFI between cell types was consistent across experiments. One representative of 5 similar experiments is shown as the Jurkat cell lines are clonal.

To model HIV latency given its rarity in primary patient samples, Jurkat cell lines were utilized. 3C9 Jurkat, containing a single integrated latent copy of non-infectious GFP-HIV that can be reactivated by TNFα (39), was either pre-stimulated with 1 ng/mL TNFα or unstimulated, pre-labeled with a deep red cell tracker and diluted 1:100 into the CFSE labeled HIV^−^ E6.1 parental Jurkat cell line to create a Jurkat mix (Figure 1A). It was observed that HIV^+^ 3C9 had a higher CD2 MFI than uninfected E6.1 parental Jurkat and that cells actively expressing GFP-HIV had the highest CD2 MFI (Figure 1B), suggesting that CD2 may indeed be a marker of HIV infection status of Jurkat cells in this study. This observation is consistent with previous findings (21) and thus alefacept may selectively target HIV^+^ 3C9 for killing in this system.

### Primary NK cells selectively target HIV^+^ jurkat for killing by ADCC with alefacept more than HIV^−^ jurkat

Healthy donor primary NK cells were co-cultured with the Jurkat mix and cytotoxicity was enumerated by absolute count flow cytometry. Preliminary experiments contained the additional control of NK cells with no antibody and no difference was observed between NK cells alone or NK cells with isotype control IgG1 (data not shown). HIV^+^ 3C9 was targeted for killing by primary NK cells and alefacept significantly more than HIV^−^ E6.1 in Jurkat mix co-cultures (Figures 1C,D; full gating strategy Figure S1). There was no significant difference between ADCC killing with alefacept when 3C9 were unstimulated or pre-stimulated with TNFα, yet higher spontaneous (IgG1) killing of 3C9 and E6.1 was observed when 3C9 was pre-stimulated with TNFα. Primary NK cells exhibit a high degree of NK cell death, which could lead to the depletion of NK cells and an attenuation of target cell killing (Figure 1D). Taken together, these data suggest that “kick and kill” HIV cure strategies may not necessarily elicit as robust ADCC toward HIV^+^ 3C9 as observed without pre-stimulation and reactivation of the virus. Additionally, there may be off target effects such as uninfected bystander cell death with a “kick and kill” approach as more spontaneous killing of HIV^−^ E6.1 was observed when 3C9 cells were pre-stimulated with TNFα in this system.

### Engagement of CD2 during NK cell expansion rescues ADCC defect observed with traditional expansion and reduces NK cell death

**Figure 2:**
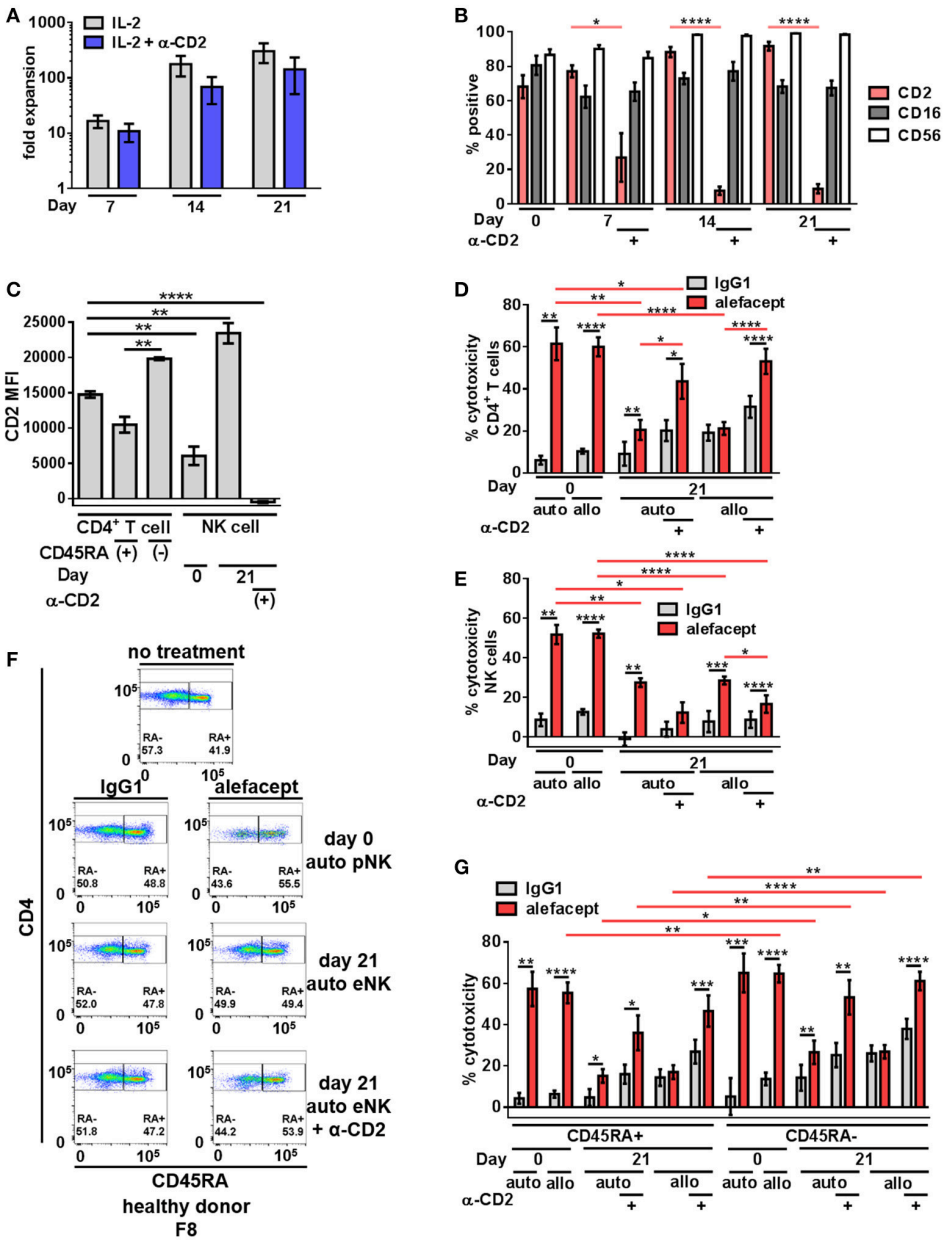
Expanded NK cells exhibit enhanced spontaneous killing and reduced ADCC compared to primary NK cells; ADCC can be rescued by engaging CD2. **(A)** Primary NK cells from healthy donors were expanded on K562 C9 feeder cells with membrane bound IL-21 with recombinant human IL-2 ± BD FastImmune^TM^ α-CD2. **(B)** Percent positive of total population surface markers CD2, CD16, and CD56 throughout NK cell expansion as measured by flow cytometry. Statistical significance between CD2 across conditions are indicated in red. **(C)** CD2 MFI of cell types in killing assay where primary NK cells (day 0) or expanded NK cells (day 21) are co-cultured with CD4^+^ T cells from the same donor (self;auto) or mismatched donor (non-self;allo) for 19 h with E:T = 1:1 and 10 μg/mL alefacept or IgG1 control antibody. **(D)** Percent cytotoxicity of self (auto) or non-self (allo) CD4^+^ T cells after co-culture with primary NK cells (day 0) or expanded NK cells (day 21) as measured by absolute count flow cytometry **(E)** Percent cytotoxicity of self (auto) or non-self (allo) NK cells (NK cell death) after co-culture with CD4^+^ T cells as measured by absolute count flow cytometry **(F)** Representative flow cytometry plots of healthy donor day 0 primary NK cells or day 21 expanded NK cells killing self (auto) CD4+ T cells with CD4 vs. CD45RA. **(G)** Percent cytotoxicity of CD4^+^ T cell subsets CD45RA+ and CD45RA– of bulk CD4^+^ T cell population shown in **(D)**. Mean and SEM shown for 4 healthy donors, *P ≤ 0.05, **P ≤ 0.01, ***P ≤ 0.001, ****P ≤ 0.0001. For percent cytotoxicity graphs, statistical significance between IgG1 and alefacept conditions are indicated with black bars and statistical significance between cell types with alefacept are indicated with red bars.

To increase NK cell numbers and attempt to decrease the NK cell death observed with primary NK cells, NK cell expansion was utilized. We hypothesized that engaging CD2 on NK cells during expansion would decrease CD2 surface expression as well as NK cell death resulting in potentially enhanced ADCC toward primary CD4+ T cells from healthy donors. Stimulation of CD2 has been shown to deliver signals for NK and T cell activation (49–51) and some NK cell activation/expansion kits (Miltenyi Biotec) include α-CD2 monoclonal antibody. To this end, primary NK cells from 4 healthy donors were expanded on K562 Cl9 mIL21 feeder cells as described previously, with or without BD FastImmune^TM^ α-CD2 which was only present during the expansion cultures and not included in downstream cytotoxicity assays (40, 47). At days 0, 7, 14, and 21 of expansion NK cells were analyzed by flow cytometry for the presence of CD2, CD16, and CD56 (Figure S2). Engaging CD2 during expansion with BD FastImmune^TM^ α-CD2 had no effect on fold expansion of NK cells (Figure 2A) but beginning on day 7, conditions that included BD FastImmune^TM^ α-CD2 during expansions significantly decreased CD2 on the cell surface as measured by percent CD2 positive and CD2 MFI while retaining CD16 and CD56 at levels similar to untreated expanded NK cells (Figures 2B,C).

Expanded NK cells and primary NK cells were used as effector cells in cytotoxicity assays against CD4+ T cells from healthy donors (Figure S3). We observed robust ADCC mediated cytotoxicity with alefacept from primary NK cells (day 0) and significantly less ADCC with alefacept from expanded NK cells (day 21) as well as increased spontaneous killing with IgG1 control from expanded NK cells at day 21. We also observed no difference in the preference of primary NK cells or expanded NK cells to kill donor-matched self CD4+ T cell targets (auto) or donor-mismatched non-self CD4+ T cell targets (allo). As expected, engaging CD2 with BD FastImmune^TM^ α-CD2 during NK cell expansion significantly increased the ADCC potential of expanded NK cells toward CD4+ T cell targets as well as reduced killing of NK cells with alefacept. However, engaging CD2 with BD FastImmune^TM^ α-CD2 during NK cell expansion also induced more spontaneous killing of CD4+ T cell targets than did primary NK cells (Figure 2D). NK cell death was also examined in the cytotoxicity assay and it was again observed that primary NK cells demonstrated a high degree of NK cell death via ADCC with alefacept but low spontaneous killing with IgG1 control (Figure 2E). Expanded NK cells had reduced death with alefacept compared to primary NK cells. This is likely not due to reduced overall ADCC cytotoxicity as engaging CD2 during expansion reduced CD2 on the NK cell surface, improved cytotoxicity toward CD4+ target cells as well as reduced cytotoxicity toward NK cells (Figure 2E).

We also sought to determine if primary or expanded NK cells demonstrated a preference to kill memory CD4+ T cells. To this end, the CD4+ T cell killing was subset into CD45RA+ and CD45RA– memory cells. We found that donor mismatched (allo) primary NK cells kill CD4+ CD45RA– memory T cells significantly more than CD4+ CD45RA+ T cells. Both auto and allo expanded NK cells had a preference to kill CD4+ CD45RA– memory T cells more than CD4+ CD45RA+ T cells (Figures 2F,G), however NK cell expansion decreased ADCC potential. Engaging CD2 during NK cell expansion rescued this defect, restoring the cytotoxicity of CD4+ CD45RA– cells to levels similar to that exhibited by primary NK cells. Additionally, we observed no difference in expanded NK cells to kill bulk allo CD4+ T cells, CD45RA+ or CD45RA– subsets between IgG1 and alefacept, suggesting that allo killing via expanded NK cells without engaging CD2 during expansion is spontaneous in nature (Figures 2D,E).

### CD16.NK-92 has natural preference to kill CD45RA– CD4^+^ memory T cells and is further enhanced by alefacept

Given limited numbers of primary NK cells and the reduced antibody-dependent cell-mediated cytotoxicity observed with expanded NK cells, the length of time it takes to expand these cells as well as evidence that NK cells from HIV^+^ individuals are hypo functional (52), a NK cell line was explored as a mediator of ADCC toward primary CD4+ T cells. NK-92 has been characterized to have most NK cell activating receptors such as NKp30 and NKp46 and lacks inhibitory KIR, making the baseline cytotoxic potential of NK-92 quite high (53). Consistently, NK-92 was shown to be highly activated and cytotoxic to a variety of cell types, however lacks CD16 to engage in ADCC (54) which has been addressed by retroviral transduction. NK-92 has been shown to be safe, well tolerated and effective in cancer clinical trials against both solid and liquid tumors (55–58). The many advantages of NK-92 cell line are that cells can be irradiated prior to infusion for maximum safety while retaining cytolytic capacity, large numbers can be grown more cost-effectively than expanding autologous or allogeneic NK cells and finally, more predictable clinical outcomes are expected secondary to master cell line homogeneity. NK-92 expressing CD16, also known as haNK^®;^, as well as CAR-expressing NK-92 (referred to as taNK^TM^) are currently in clinical trials: NCT03027128, NCT03329248, NCT03387085, NCT03387111, NCT03387098, NCT03383978. CD16+ NK-92 cells have been shown to participate in robust ADCC against a spectrum of tumor cells (59). The CD16.NK-92 used here co-expresses the high affinity variant of CD16 (176 V) as well as GFP, lacks inhibitory KIR, and is reliant on exogenous IL-2 (41, 42).

**Figure 3:**
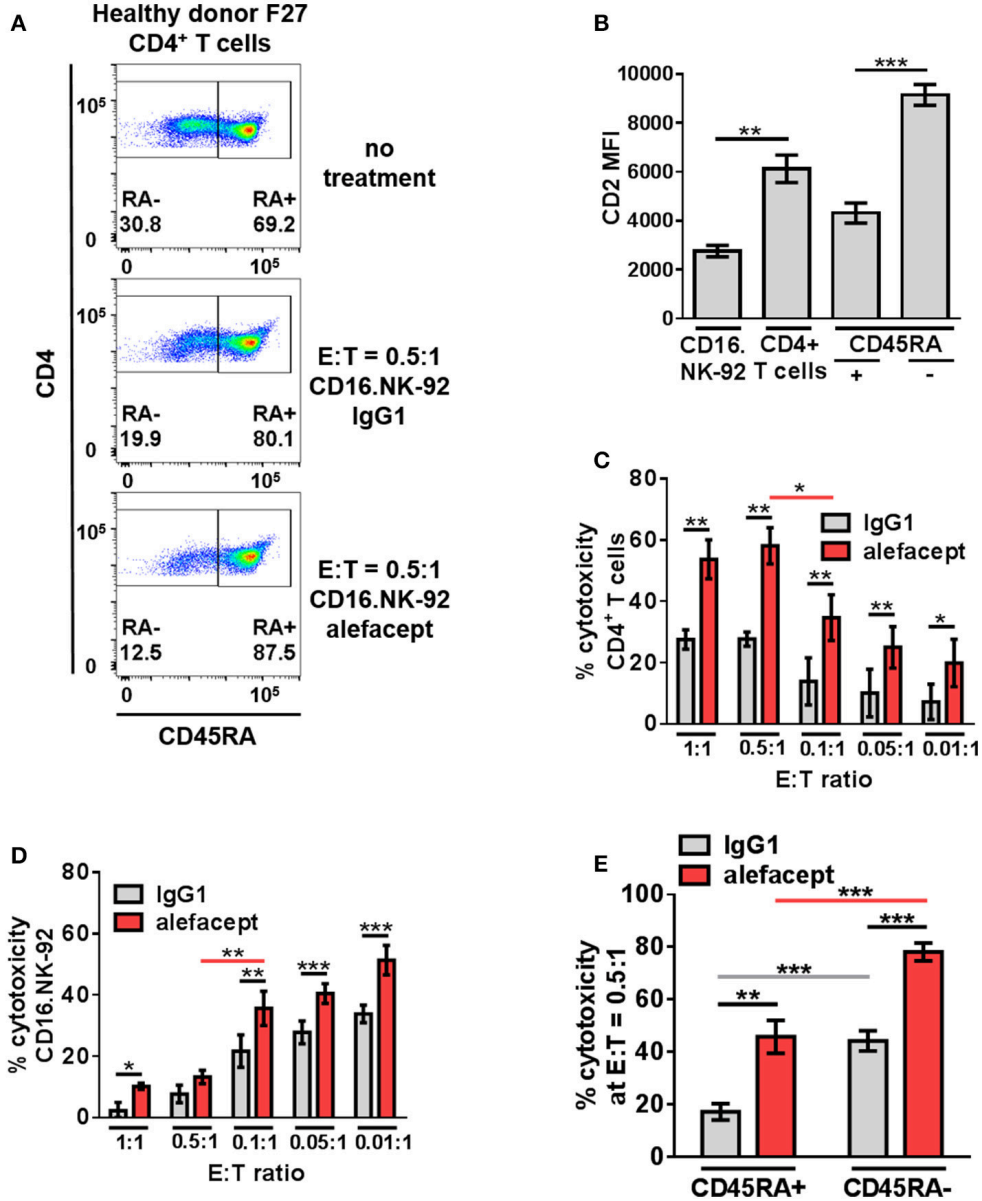
CD16.NK-92 has natural preference to kill CD45RA– CD4^+^ memory T cells and that is enhanced by alefacept in healthy donors. **(A)** Representative healthy donor flow cytometry plots from killing assay with CD16.NK-92 and healthy donor CD4^+^ T cells 19 h in co-culture with CD45RA subsetting shown with no treatment, with CD16.NK-92 and 10 μg/mL alefacept or IgG1 control antibody. **(B)** CD2 MFI of CD16.NK-92, and CD4^+^ T cells. **(C)** Percent cytotoxicity of CD4^+^ T cells in killing assay with CD16.NK-92 after 19 h in co-culture with 10 μg/mL alefacept or IgG1 control antibody and E:T ratio dose response, as measured by absolute count flow cytometry. **(D)** Percent cytotoxicity of CD16.NK-92 in killing assay 19 h in co-culture with 10 μg/mL alefacept or IgG1 control antibody and E:T ratio dose response, as measured by absolute count flow cytometry. **(E)** Percent cytotoxicity of CD45RA+ and CD45RA– subsets of CD4^+^ T cells (as shown in **C**) at E:T = 0.5:1 after 19 h co-culture with CD16.NK-92 and 10 μg/mL alefacept or IgG1 control antibody, as measured by absolute count flow cytometry. Mean and SEM shown for 6 healthy donors, *P ≤ 0.05, **P ≤ 0.01, ***P ≤ 0.001. Statistical significance between IgG1 and alefacept conditions are indicated with black bars, statistical significance between cell types with IgG1 are indicated with gray bars and statistical significance between cell types with alefacept are indicated with red bars.

In addition to a dose response of alefacept in which 10 μg/mL was determined to be the optimal concentration for use *in vitro* (Figure S4A), an effector cell dose response was performed using CD16.NK-92 E:T ratios ranging from 1:1 to 0.01:1. Low E:T ratios were explored to minimize spontaneous killing (IgG1) and NK-92 has been previously shown to be effective at low E:T ratios (60). CD16.NK-92 has a lower CD2 MFI than healthy donor CD4+ T cells (Figure 3B). Indeed, it was revealed that at lower E:T ratios there was less spontaneous killing in the presence of IgG1, however the greatest difference in cytotoxicity between alefacept and IgG1 was observed at E:T = 0.5:1 (Figures 3A,C) as well as the least amount of CD16.NK-92 cell death (Figure 3D). At E:T = 0.1:1 there was significantly less killing of CD4+ T cell targets than at E:T = 0.5:1 and NK cell death also increased significantly at E:T = 0.1:1 (Figures 3C,D). For this reason, E:T = 0.5:1 was used for the remainder of studies with CD16.NK-92 as cytotoxic effector cells. Additionally, we have explored the experimental condition of CD16.NK-92 effector cells + CD4+ T target cells with no antibody and observed similar cytotoxicity with no antibody as with isotype control IgG1 (Figure S4A). As IgG1 can bind Fc receptor CD16, we have chosen to use the isotype of alefacept, IgG1, as the spontaneous killing control (43–45). CD16.NK-92 was also evaluated for the presence of CD16 and GFP prior to set up of ADCC co-culture (Figure S4B).

Killing of CD4+ T cell subsets by CD16.NK-92 was further divided into CD45RA– memory T cells and CD45RA+ which contains mostly naïve CD4+ T cells. When CD16.NK-92 and CD4+ T cells are co-cultured together there was an intrinsic preference for CD16.NK-92 to significantly kill CD45RA– memory T cells spontaneously with IgG1 more than CD45RA+ cells. ADCC with alefacept further enhanced the preference to kill CD45RA– memory T cells, which have the highest CD2 MFI (Figures 3A,B,E). To confirm that CD16.NK-92 can still kill target cells via ADCC with alefacept after being irradiated, as per clinical indications prior to infusion, an additional killing assay was performed with 10 Gy γ-irradiated CD16.NK-92 and 4 healthy donor CD4+ T cells. Consistent with previous findings, 10 Gy irradiated CD16.NK-92 can still kill target cells via ADCC and the preference for CD45RA– memory T cells is preserved [Figure S5; (58, 59, 61)].

### CD16.NK-92 and alefacept combination significantly depletes the CD45RA– CD27^+^ central memory/transitional memory (T_CM/TM_) subset in healthy donor CD4^+^ T cells

**Figure 4:**
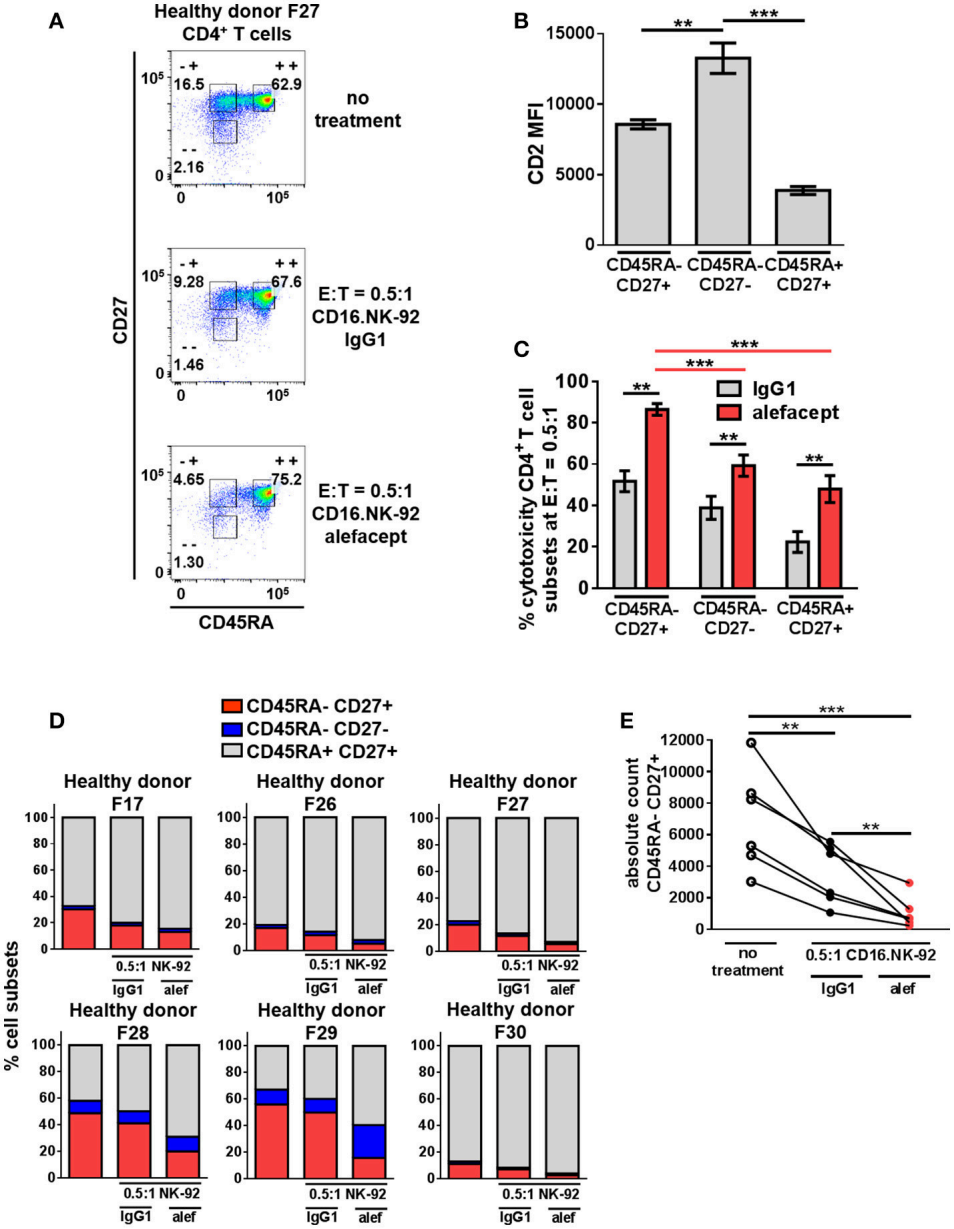
CD45RA– CD27+ Central Memory/Transitional Memory (T_CM/TM_) CD4^+^ T cell subset is significantly depleted by CD16.NK-92 and alefacept in healthy donors. **(A)** Representative flow cytometry plots of CD16.NK-92 killing of CD4^+^ T cell subsets in healthy donor at E:T = 0.5:1, 19 h co-culture with 10 μg/mL alefacept or IgG1 control antibody. **(B)** CD2 MFI of CD4^+^ T cell subsets. **(C)** Percent cytotoxicity of CD4^+^ T cell subsets (same CD4^+^ T cell donors from Figure 3) co-cultured with CD16.NK-92 for 19 h at E:T = 0.5:1 with 10 μg/mL alefacept or IgG1 control antibody, as measured by absolute count flow cytometry. **(D)** Percent of whole representations of CD4^+^ T cell subsets by donor with and without treatment. **(E)** Absolute count of CD27+ CD45RA+ T_CM/TM_ subset with no treatment and with CD16.NK-92 at E:T = 0.5:1 and 10 μg/mL alefacept or IgG1 control antibody after 19 h in co-culture. Mean and SEM are shown for 6 healthy donors, **P ≤ 0.01, ***P ≤ 0.001. For percent cytotoxicity graph, statistical significance between IgG1 and alefacept conditions are indicated with black bars and statistical significance between cell subsets with alefacept are indicated with red bars.

The same cytotoxicity assay of CD16.NK-92 killing CD4+ T cells from healthy donors was further subset into memory T cell populations at E:T = 0.5:1 (Figure 4A and Figure S6). Although the CD45RA– CD27– effector memory (T_EM_) subset had the highest CD2 MFI (Figure 4B), the CD45RA– CD27+ central memory/transitional memory subset (T_CM/TM_) was subject to the highest percent cytotoxicity with alefacept (Figure 4C). When each CD4+ T cell donor subset was represented as part of the whole or by absolute numbers, a reduction of CD45RA– CD27+ (T_CM/TM_) was observed that is significant both with spontaneous killing by CD16.NK-92 with IgG1 and additive with ADCC killing with alefacept (Figures 4D,E). CD27- CD45RA+ representing terminally differentiated (T_TD_) CD4+ T cell subset was excluded from analysis as it was an extremely small population in each donor that increased when CD16.NK-92 was added to the co-culture.

### CD16.NK-92 and alefacept combination also targets the CD45RA– CD27^+^ T_CM/TM_ CD4^+^ T cell subset in HIV^+^ donor CD4^+^ T cells

**Figure 5:**
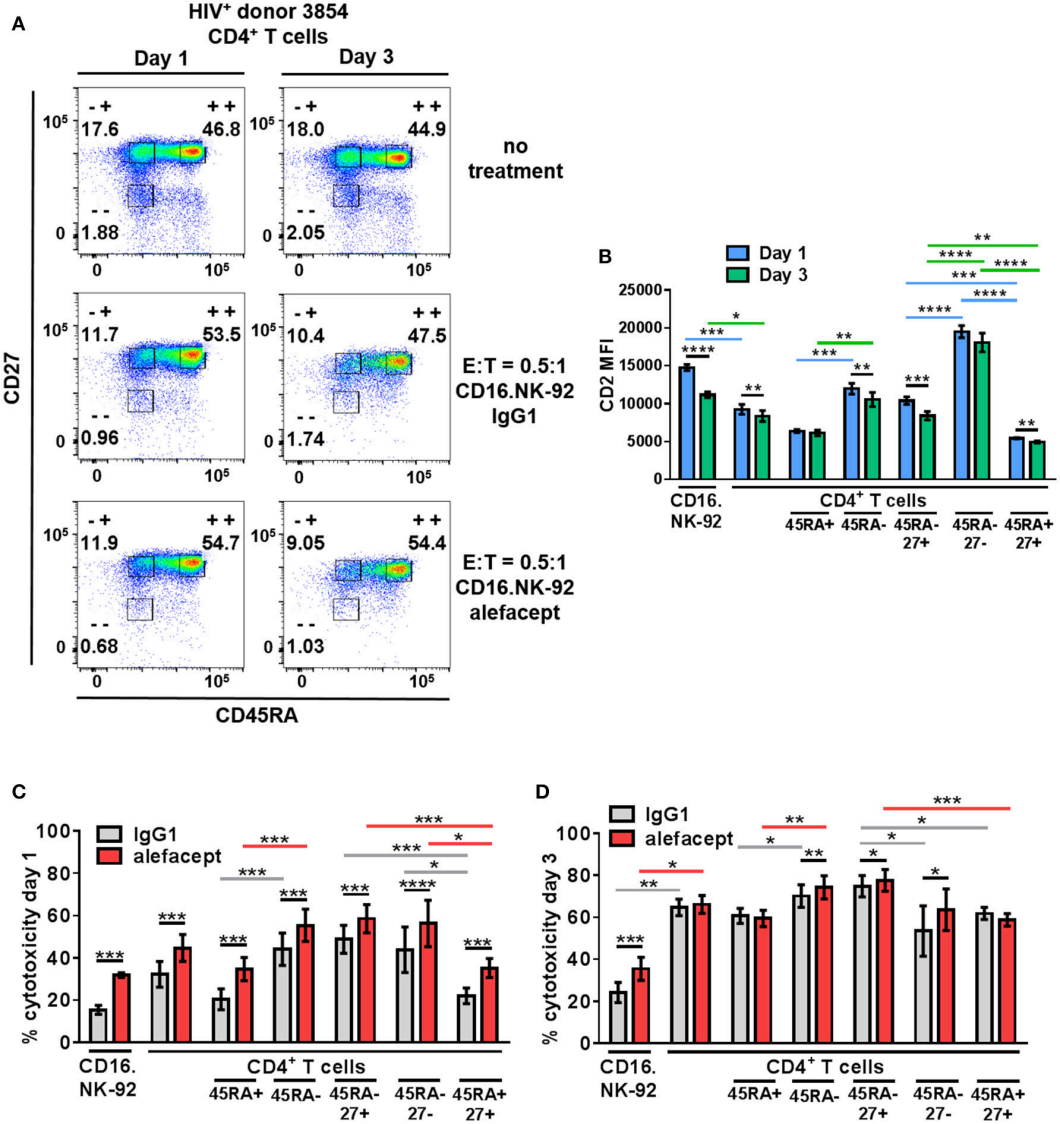
CD16.NK-92 and alefacept also target CD45RA– CD27+ T(_CM/TM_) CD4^+^ T cell subset in HIV^+^ donors. **(A)** Representative flow cytometry plots of HIV^+^ CD4^+^ T cell donor with memory subsetting at 19 h (1 day) and 3 days with no treatment, with CD16.NK-92 and 10 μg/mL alefacept or IgG1 control antibody at E:T = 0.5:1. **(B)** CD2 MFI of all cell types and subsets in co-culture at days 1 and 3. **(C)** Percent cytotoxicity of CD16.NK-92, CD4^+^ T cells, and CD4^+^ T cell subsets in HIV^+^ donors after co-culture for 19 h (1 day) with 10 μg/mL alefacept or IgG1 control antibody at E:T = 0.5:1 as measured by absolute count flow cytometry. **(D)** Percent cytotoxicity of CD16.NK-92, CD4+ T cells, and CD4+ T cell subsets after co-culture for 3 days with 10 μg/mL alefacept or IgG1 control antibody at E:T = 0.5:1 as measured by absolute count flow cytometry. Mean and SEM of 6 HIV^+^ donors shown, *P ≤ 0.05, **P ≤ 0.01. Statistical significance CD2 MFI at days 1 and 3 of the same cell type are indicated with black bars, statistical significance between cell types and subtypes of CD2 MFI at day 1 are indicated with blue bars and at day 3 by green bars. Statistical significance between IgG1 and alefacept conditions are indicated with black bars, statistical significance between cell types with IgG1 are indicated with gray bars and statistical significance between cell types with alefacept are indicated with red bars.

To recapitulate our findings in HIV^+^ primary patient samples, cytotoxicity assays were set up at E:T = 0.5:1 with CD16.NK-92 and CD4+ T cells isolated from leukapheresed HIV^+^ PBMCs from 6 patients treated with ART and identified as having a controlled viral load. Co-cultures were set up for 3 days with a sample taken at day 1 to enumerate cytotoxicity by flow cytometry at two time points (Figure 5A). At day 3, the co-cultures were also harvested for nucleic acid isolation and qPCR as well as flow cytometry. It was observed that the CD2 MFI of CD16.NK-92 was higher than normally observed, and in fact higher than the CD2 MFI of CD4+ T cell targets (Figure 5B). This could be due to using a late passage culture of CD16.NK-92 and further influenced by recent feeding of cells with IL-2. However, increased CD16.NK-92 CD2 MFI did not result in greater NK-92 cell death, as this was consistently lower than the killing of target cells (Figures 5C,D) at both days 1 and 3. At day 1, there is a preference to kill CD45RA– memory T cells from HIV^+^ donors spontaneously (IgG1) over CD45RA+ and this is enhanced with alefacept, as we have previously observed in healthy donors. However, at day 1, there is somewhat equal killing of CD45RA– CD27+ T(_CM/TM_) and CD45RA– CD27– T(_EM_) both spontaneously (IgG1) and via ADCC (alefacept). Naïve CD45RA+ CD27+ (T_N_) subset is killed the least by CD16.NK-92, both spontaneously and with alefacept and also has the lowest CD2 MFI (Figures 5B,C). By day 3, it appeared that the killing became saturated as spontaneous (IgG1) and ADCC (alefacept) killing was similar in each subset. However, even at saturating conditions the CD45RA– CD27+ T(_CM/TM_) subset was subject to spontaneous killing by CD16.NK-92 significantly more than CD45RA– CD27- T(_EM_) subset (Figure 5D).

### CD16.NK-92 and alefacept combination significantly reduce HIV DNA *in vitro* from primary patient samples

**Figure 6:**
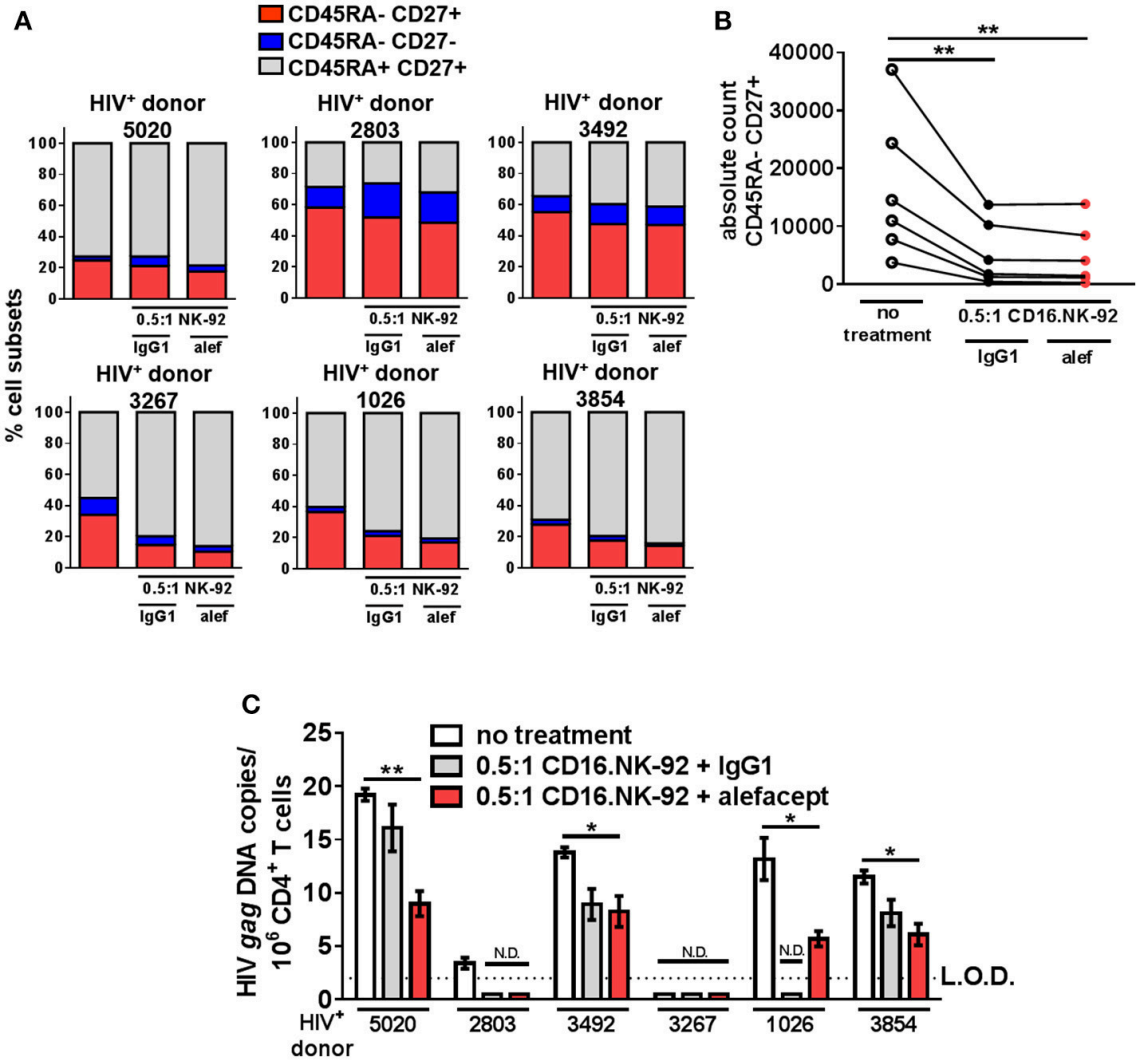
CD16.NK-92 and alefacept significantly reduce HIV DNA *in vitro*. **(A)** Percent of whole representation of CD4+ T cell memory subsets by HIV^+^ donor with no treatment, after 3 day co-culture with CD16.NK-92 and 10 μg/mL alefacept or IgG1 control antibody. **(B)** Absolute counts of CD27+ CD45RA– T(_CM/TM_) subset in HIV^+^ donors after 3 days in co-culture with CD16.NK-92 and 10 μg/mL alefacept or IgG1 control antibody. **(C)** HIV DNA copies per million CD4^+^ T cells as measured by quantitative PCR, with no treatment or after 3 day co-culture with CD16.NK-92 and 10 μg/mL alefacept or IgG1 control antibody. Samples in which HIV *gag* DNA was not detectable are indicated with N.D. Dotted line represents the limit of detection (L.O.D.) of 2 copies of HIV *gag* DNA copies/10^6^ CD4+ T cells. Mean and SEM shown for 3 qPCR reactions of same sample and 6 HIV_+_ donors, *P ≤ 0.05, **P ≤ 0.01.

By day 3 of the HIV^+^ donor CD4+ T cell killing assay, the spontaneous cytotoxicity exhibited by CD16.NK-92 increased to the level of killing with alefacept and this was reflected in both the part of whole analysis for each donor as well as the absolute count of CD45RA– CD27+ T(_CM/TM_) subset (Figures 6A,B). To discern if there was a reduction of HIV DNA, qPCR for HIV *gag* was performed on the remaining cells after 3 days co-culture with CD16.NK-92 and normalized with the housekeeping gene RPP30. Data was fit to a standard curve utilizing ACH2 as the spike-in with a constant background of uninfected cellular nucleic acid in which 1 copy of HIV *gag* could be readily detected (Figure S7B). Some experimental samples were not determined as they fell below the limit of detection of our assay (N.D.) of 1 copy HIV *gag* per well (Figures S7A,C). However, samples with detectable HIV *gag* showed significant decreases in HIV *gag* when treated with CD16.NK-92 and alefacept (Figure 6C). In some donors, the copies of HIV *gag* were similar between IgG1 control and alefacept, providing additional evidence that CD16.NK-92 was effective at reducing HIV DNA alone and that NK cytotoxicity was likely saturated by day 3 consistent with our flow cytometry data (comparing Figure 5C with Figure 5D). RT-qPCR was also utilized to detect HIV RNA, however all experimental samples were below the limit of detection (data not shown). Taken together, our data demonstrates that, while spontaneous killing of well-controlled HIV^+^ primary patient CD4+ T cells by CD16.NK-92 is quite high by day 3, the reduction of HIV *gag* DNA frequency was still significant and further augmented by alefacept for some donors, suggesting that a combinatorial approach utilizing CD16.NK-92 and alefacept may add value to ongoing strategies to reduce the latent HIV reservoir.

## Discussion

Utilizing highly sensitive and quantitative flow cytometry to enumerate and characterize the remaining live cells after co-culture with cytotoxic NK cells and alefacept has allowed us to show as a proof of concept that NK cells and alefacept can be combined to selectively target latently infected HIV^+^ cells *in vitro*. We have shown a preference for primary NK cells from healthy donors to target latently HIV infected 3C9 Jurkat for killing over uninfected E6.1 Jurkat in a Jurkat mix co-culture. Consistent with previous findings that HIV-1 latently infected memory CD4+ T cells express high levels of CD2 (21), our results show that latently infected 3C9 Jurkat has a higher CD2 MFI than uninfected parental Jurkat E6.1. Similarly, when HIV-GFP is expressed, CD2 MFI also increases further; however reactivation of the virus is not necessary for optimal killing by primary NK cells in this system. Interestingly, reactivation of HIV-GFP in 3C9 resulted in non-specific killing of uninfected E6.1 cells supporting the concern that latency reversing agents and related “kick and kill” HIV cure strategies may have deleterious effects.

We have also demonstrated a role for CD2 in NK cell ADCC. Specifically, rescue of an ADCC defect was observed with expanded NK cells treated with BD FastImmune^TM^ α-CD2. We hypothesized that BD FastImmune^TM^ α-CD2 would reduce NK cell death observed with primary and expanded NK cells, and discovered that engaging CD2 during NK cell expansion both reduced NK cell death as well as increased target cell killing. Although engaging CD2 on an NK cell during NK cell expansion rescued the ADCC defect observed without CD2 engagement, cytotoxicity was not restored to levels observed with primary NK cells. The mechanism of CD2 engagement leading to rescue of ADCC remains to be elucidated and is likely involved in the activation status of the cell.

While exploring different NK cell-based approaches to mediate ADCC with alefacept, we found that CD16.NK-92 showed a natural preference to kill CD45RA– memory CD4+ T cells without alefacept. CD4+ memory T cells are thought to be the longest-lived seat of the latent HIV reservoir and are capable of multiple cellular divisions (3, 62–67). Combining this natural preference for CD4+ memory T cell killing by CD16.NK-92, which is further enhanced by adding alefacept, we were able to show that although the CD45RA– CD27– T(_EM_) subset exhibits the highest CD2 MFI, fortuitously the CD45RA– CD27+ T(_CM/TM_) subset was the most targeted in both healthy and HIV^+^ CD4+ T cell donor primary cells, perhaps due to CD16.NK-92 sensing via an unknown mechanism. Thus, all memory CD4+ T cell subsets were selectively targeted greater than their naïve counterparts and the longest lived memory subsets appeared to be most selected for killing. Alternatively, this could be interpreted as naïve cell resistance to killing rather than specific targeting of memory cells.

Additionally, in HIV^+^ donor CD4+ T cells we observed a significant reduction of HIV *gag* DNA by adding CD16.NK-92 and alefacept over 3 days. With the caveat that the spontaneous cytotoxicity with IgG1 nearly reached the level achieved with alefacept at day 3 when the co-cultured cells were collected for qPCR analysis, we still observed an additional decrease in HIV *gag* DNA with alefacept in some donors. We have consistently observed across many experiments that ~80–90% cytotoxicity appears to be the upper limit of this *in vitro* system. When cytotoxicity reaches this limit, specificity is often lost and thus relatively, spontaneous killing increases. Given that NK cells in chronic virally infected patients such as HIV are often hypo functional or dysfunctional (52), CD16.NK-92 appears well poised for clinical application in decreasing the HIV reservoir. Perhaps due to the lack of inhibitory KIRs and presence of many activating receptors, CD16.NK-92 appears to seek and destroy HIV^+^ cells *in vitro* and this is enhanced in the presence of alefacept. Our *in vitro* results also indicated that alefacept can be administered effectively without the need of latency reversing agents to remove cells bearing HIV provirus from donors well-controlled to undetectable levels with ART.

Our data with HIV^+^ donor CD4+ T cells (with in most cases undetectable HIV viremia for several years) suggest that CD2 may be used to identify the HIV reservoir in some individuals, as alefacept is able to functionally target and decrease these cell populations. In other donors, the observed cytotoxicity and reduction of HIV *gag* DNA appears less dependent on CD2 and CD16.NK-92 played a larger role by spontaneous killing. Taken together with our latent Jurkat mix model and increased CD2 MFI observed in both resting and activated HIV-GFP+ 3C9, we remain hopeful that CD2 may be used to identify the latent HIV infected reservoir in some individuals consistent with previous findings identifying CD2 as a marker of the latent HIV reservoir in resting CD4+ T cells (21). We caveat our findings by noting that CD2 may be an indirect marker of HIV infection and instead a marker of cell activation. As such, CD2 may not be a true marker of the latent HIV reservoir in memory CD4+ T cells. It is not known why CD2hi enriched for T cells bearing the HIV reservoir better than other activation markers when compared by the Romerio lab. However, empirically, PBMCs sorted for CD2hi from HIV+ individuals well-controlled on ART possessed the majority of the inducible HIV reservoir (21). This population of CD2hi cells is the same population depleted in patients by alefacept (29–37). Concomitantly in our experiments, it could be interpreted that the targeting and elimination of HIV^+^ cells is an effect of CD16.NK-92 and alefacept depletion of CD2 hi CD4+ memory T cells where HIV is likely to reside and not a specific targeting and killing of HIV^+^ cells. We believe that this approach may indeed prove effective *in vivo*. While leaving the patient temporarily immunosuppressed, it may be necessary to purge memory T cells, some of which contain HIV, in order to significantly reduce the longest lived HIV reservoir in CD4+ T cells. The subsets of memory T cells can then be re-established over time from largely untouched naïve T cells which have a lower frequency of HIV infection, in part likely due to lower levels of the HIV co-receptor CCR5 (64, 68–73). Conceptually, this intervention with alefacept has been used extensively & successfully in psoriasis patients whose immune system is dysfunctional by definition. In short, alefacept therapy is the only therapy to date amongst the numerous psoriasis therapies which has conferred durable remission (74). Likewise, the depletion of CD2hi cells by alefacept in type I diabetes patients has resulted in preservation of C-peptide (75), consistent with sustained clinical and immunological effects seen in these patients by the same T1DAL investigators (76). Depletion of CD2hi cells by alefacept has resulted in remission of other less common diseases as well such as cutaneous T cell lymphoma (77). Therefore, we hypothesize that an intervention with alefacept, augmented by CD16.NK-92 which we report here for the first time, may also have potential to preferentially kill T cells bearing HIV over HIV^−^ cells and may similarly benefit HIV^+^ individuals well-controlled on ART. Ultimately, we hypothesize that these interventions may preferentially decrease the number of T cells bearing HIV and thus extend ART-free periods without detectable viral recrudescence. We are evaluating this possibility with HIV^+^ humanized mouse models and SHIV-infected macaques.

In addition to CD2, CD32 has also been identified as a marker of the latent HIV reservoir, however no groups have been able to confirm these initial findings (13). A follow up study demonstrated that CD32 was predominantly expressed on transcriptionally active CD4+ T cells, not resting memory T cells as previously proposed (14). Additional studies indicated that although CD32+ CD4+ T cells can contain HIV DNA, CD32 is not a specific marker of the HIV reservoir (15–20). PD-1, TIGIT and LAG-3 have also been identified as novel markers enriched in latently infected HIV^+^ cells, and triple positivity highly enriches for integrated HIV DNA in patients treated with ART (78). In this study, we have chosen to target CD2 with FDA approved alefacept as this drug has had more than 20 years of experience in humans, including pregnancy, and has an excellent safety profile. Additional work is needed exploring the relationship of surface markers implicated in HIV reservoir phenotyping, e.g. CD2, CD32, PD-1, TIGIT and LAG-3. Perhaps combining biologics against these targets in addition to alefacept, or developing novel bifunctional antibodies, will provide more specificity and the HIV reservoir can be targeted more specifically with less bystander cell death and thus improve on the limitations of CD2 alone to enrich for the latent HIV reservoir in memory CD4+ T cells.

Additionally, it has been shown that T cells with high levels of intracellular CD2 also express high levels of restriction factors such as SAMHD1, p21 and SerinC5 while T cells low in intracellular CD2 express lower levels of restriction factors and are more positive for HIV-1 p24 in both untreated viremic controllers and progressors. However, when the authors looked at ART treated HIV patients, the percentage of HIV-1 p24 is actually higher in CD2+ cells in two out of three patients (79). This evidence also supports our overall hypothesis and associated goal to repurpose alefacept, a human α-CD2 monoclonal fusion protein, to target CD2hi cells to preferentially decrease the latent HIV reservoir in CD2hi memory CD4+ T cells in patients as they remain on antiretroviral therapy. These findings also provide evidence of patient variability that may require a personalized medicine approach in which patients could be screened prior to therapy to identify if they would be a candidate for alefacept therapy to decrease their unique HIV reservoir if it is indeed enriched in CD2hi cells on an individualized basis.

The question remains, is CD2 a true marker of the reservoir? If so, does HIV preferentially infect CD2hi cells or does CD2 become upregulated after infection? Additionally, why does CD16.NK-92 have a natural preference to eliminate HIV^+^ cells? The predominant mechanism of NK-92 cytotoxicity has been identified as the induction of apoptosis and/or necrosis through the perforin and granzyme pathway. NK-92 cells have more perforin and granzyme on a per cell basis than primary NK cells. NK-92 also has Fas, TWEAK, TRAIL and other surface markers that contribute to cytotoxicity via pathways other than perforin/granzyme. CD3ξ is also present intracellularly in NK-92 and likely contributes to the functionality of transduced CD16 (53). Investigation into these questions and others will be addressed as this research field progresses.

In addition to the largest and longest lived HIV reservoir in CD4+ memory T cells, there are other clinically relevant reservoirs of HIV including monocytes, tissue resident macrophages, astrocytes and microglia (80–85). It has been questioned if astrocytes are actually infected with HIV or if they have acquired integrated HIV DNA due to engulfing an infected cell (86, 87). Regardless, treatment with alefacept alone would not deplete these reservoirs as the cells do not express CD2. Our approach specifically focuses on targeting the largest, longest lived and best characterized HIV reservoir residing in CD4+ memory T cells and we acknowledge that alefacept alone is almost certainly not going to be sufficient to eliminate the HIV reservoir. However, any decrease in HIV reservoir and associated delay in HIV rebound after analytic treatment interruption within the context of an approved clinical trial would be a significant advance.

It may seem counterintuitive to treat a well-controlled ART treated and stabilized HIV^+^ patient with cytotoxic NK-92 cells and CD4+ T cell depleting antibody such as alefacept, however numerous previous studies with irradiated NK-92 as well as alefacept demonstrate excellent safety profiles (35, 55–58, 88, 89). Since *in vitro* cytotoxicity saturates at ~80%, spares many naïve CD4+ T cells and specifically reduces HIV *gag* DNA, we expect that not all CD4+ T cells will be depleted, and the patient will reconstitute their CD4+ T cell counts from uninfected naïve cells over time as has been observed clinically since 2002 with the use of alefacept in psoriasis patients. Additionally, we expect that HIV^+^ cells will not be the only cells eliminated by a CD2 targeting approach such as alefacept. Any CD2+ cells including CD8+ T cells have the potential to be compromised by this therapy. However not all cells will perish and immune function can be restored after therapy from hematopoietic stem cells as well as differentiation of naïve cells into memory cells. Our hope is that the strategy of purging the largest and longest-lived seat of the HIV reservoir, CD4+ memory T cells that are CD2hi, will reduce the HIV reservoir in some individuals such that a functional cure, for some finite period of time, in the absence of antiretroviral therapy may be possible. To put into context, far more broad reaching cytotoxic compounds such as alemtuzumab have been administered to HIV^+^ individuals (90–94). We reason that a more preferential agent such as alefacept that does not target as broadly as chemotherapeutic agents may be safer and better tolerated *in vivo*. More work is needed to provide additional proof of concept that CD16+ NK-92 and alefacept can safely reduce the HIV reservoir *in vivo* using animal models. Thus, we remain hopeful that these previously unrelated therapies can be combined to combat HIV, one of the most challenging diseases of modern history.

## Ethics statement

All patients gave informed consent according to a protocol approved by the local Case Western Reserve University Internal Review Board in addition to the American Red Cross Internal Review Board for samples acquired thereof.

## Author contributions

AT and DP conceived and designed the experiments as well as wrote the manuscript. AT and IR-G performed the experiments and analyzed the data. KC provided pertinent discussion and technical support. AT, IR-G, KC, and DP provided critiques and edited the manuscript. DP supervised the research project.

### Conflict of interest statement

The authors declare that the research was conducted in the absence of any commercial or financial relationships that could be construed as a potential conflict of interest.
