# Supplementary material for: In vitro Evidence That Combination Therapy With CD16-Bearing NK-92 Cells and FDA-Approved Alefacept Can Selectively Target the Latent HIV Reservoir in CD4+ CD2hi Memory T Cells
Source: Front Immunol. 2018 Nov 5;9:2552. doi: 10.3389/fimmu.2018.02552 (PMC6230627; doi:10.3389/fimmu.2018.02552)
Supplement: Supplementary file 1 [file Data_Sheet_1.docx]

*Supplementary Material*

***In Vitro* Evidence that Combination Therapy with CD16-bearing NK-92 cells and FDA-Approved Alefacept can Selectively Target the Latent HIV Reservoir in CD4+ CD2hi Memory T Cells**

Tomalka AG^1^, Resto-Garay I^1^, Campbell KS^2^, Popkin DL^1^*

^1^Department of Dermatology, Case Western Reserve University School of Medicine, Cleveland, OH, USA

^2^Blood Cell Development and Function Program, Institute for Cancer Research, Fox Chase Cancer Center, Philadelphia, PA, USA

*Correspondence:

Dr. Daniel Popkin

[daniel.popkin@case.edu](mailto:daniel.popkin@case.edu)


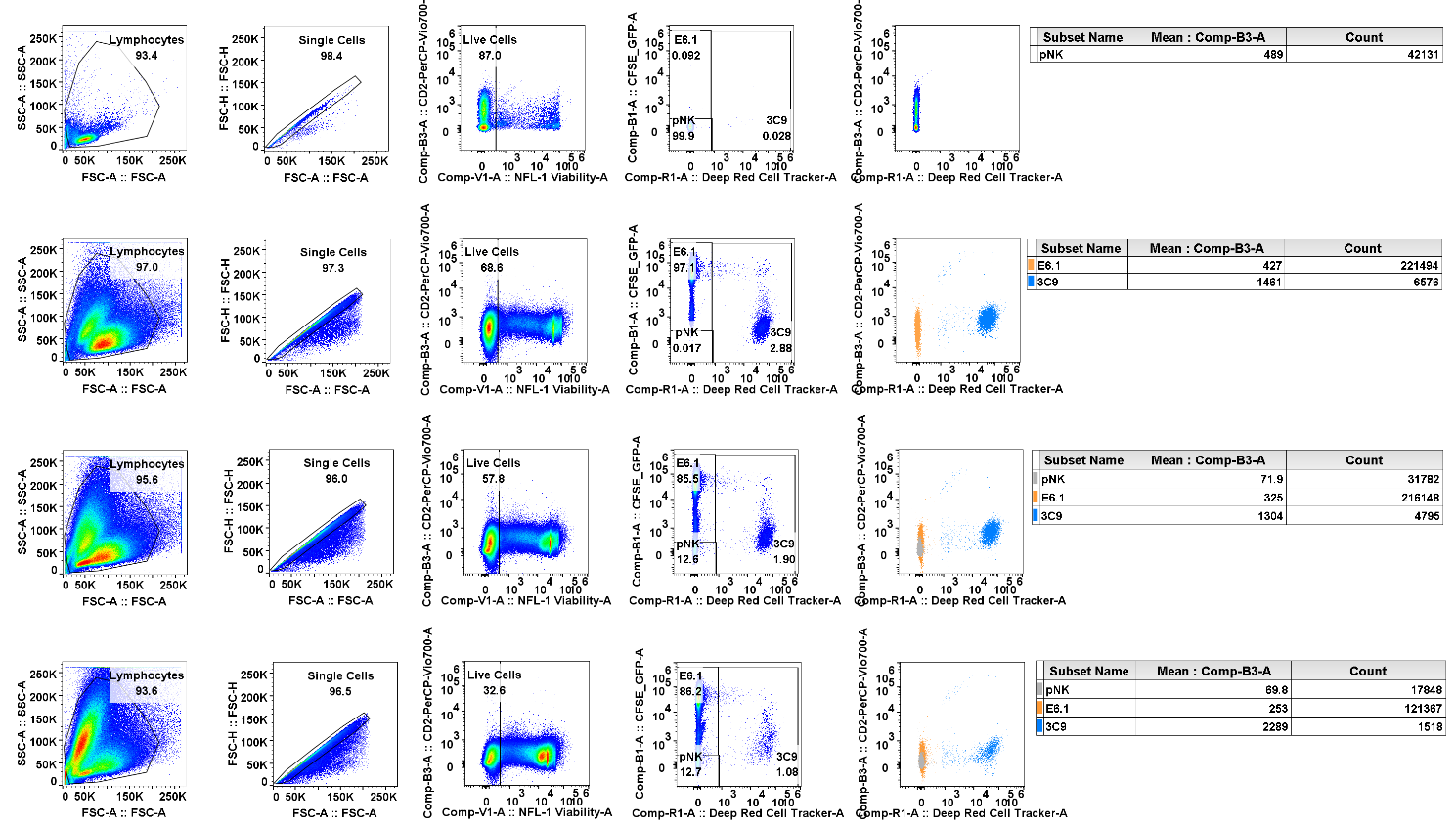


**Healthy donor F12**

**primary NK cells**

**Jurkat Mix**

**Jurkat Mix**

**+ healthy donor F12 primary NK cells**

**+ 10 µg/mL IgG1**

**Jurkat Mix**

**+ healthy donor F12 primary NK cells**

**+ 10 µg/mL alefacept**

**Figure S1. Gating strategy for primary NK and Jurkat Killing Assay.** Healthy donor primary NK cells (n = 6) were co-cultured for 19 hours at E:T = 0.1:1 with GFP-HIV^+^ 3C9 Jurkat diluted 1:100 into uninfected parent E6.1 Jurkat cell line.


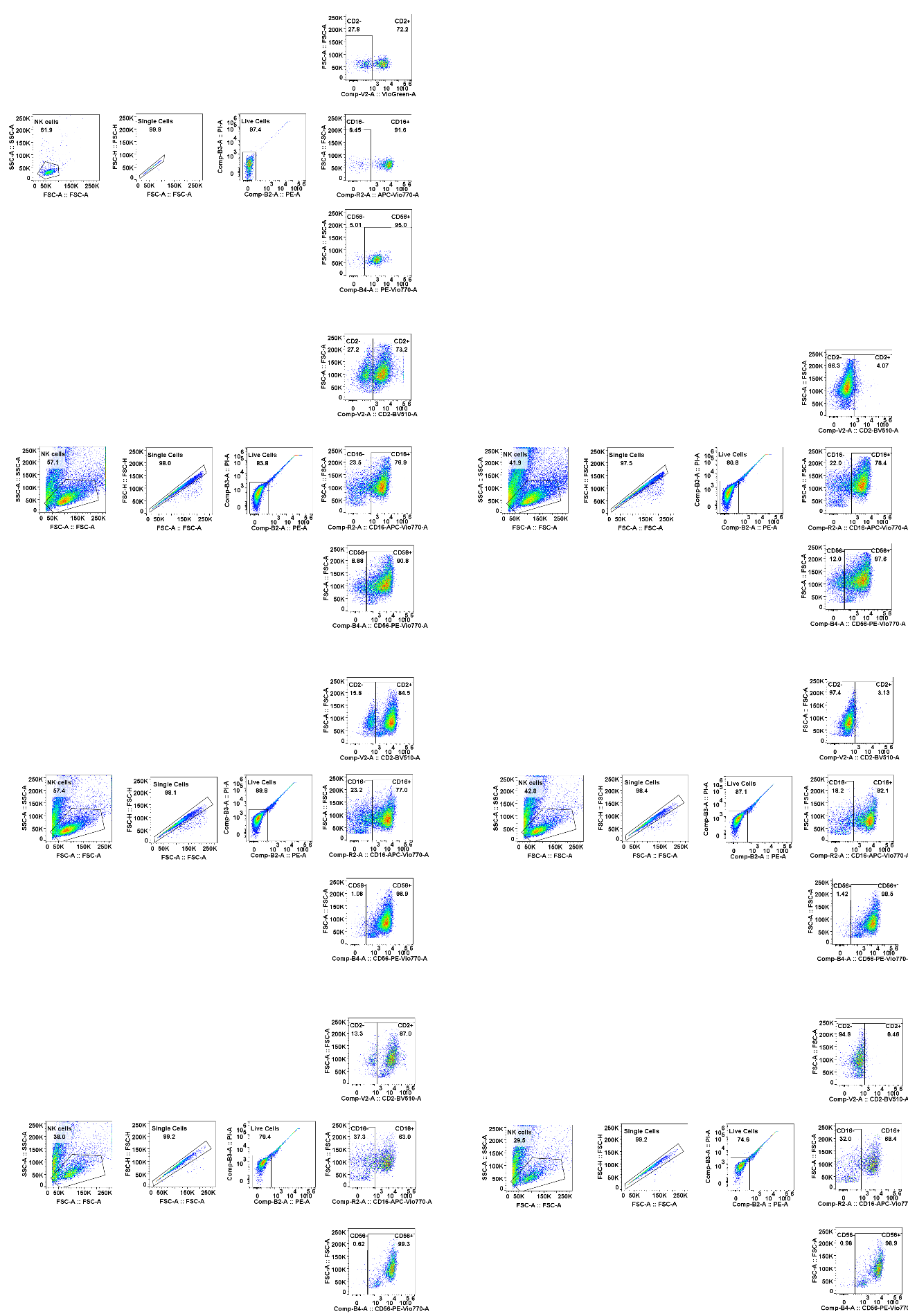


**Day 0**

**primary NK**

**Day 7**

**expanded NK**

**Day 14**

**expanded NK**

**Day 21**

**expanded NK**

**Healthy donor F7 NK cells**

**+ IL-2**

**+ BD FastImmune^TM^ α-CD2**

**+ IL-2**

**Figure S2. Gating strategy for expanded NK surface staining.** Primary NK cells from 4 healthy donors were expanded on K562 C9 feeder cells for 21 days with IL-2 and/or BD FastImmune^TM^ α-CD2. Surface markers CD2, CD16 and CD56 were monitored over time.

**Healthy donor F8 NK cells (day 0 primary or day 21 expanded)**

**+ auto CD4^+^ T cells**


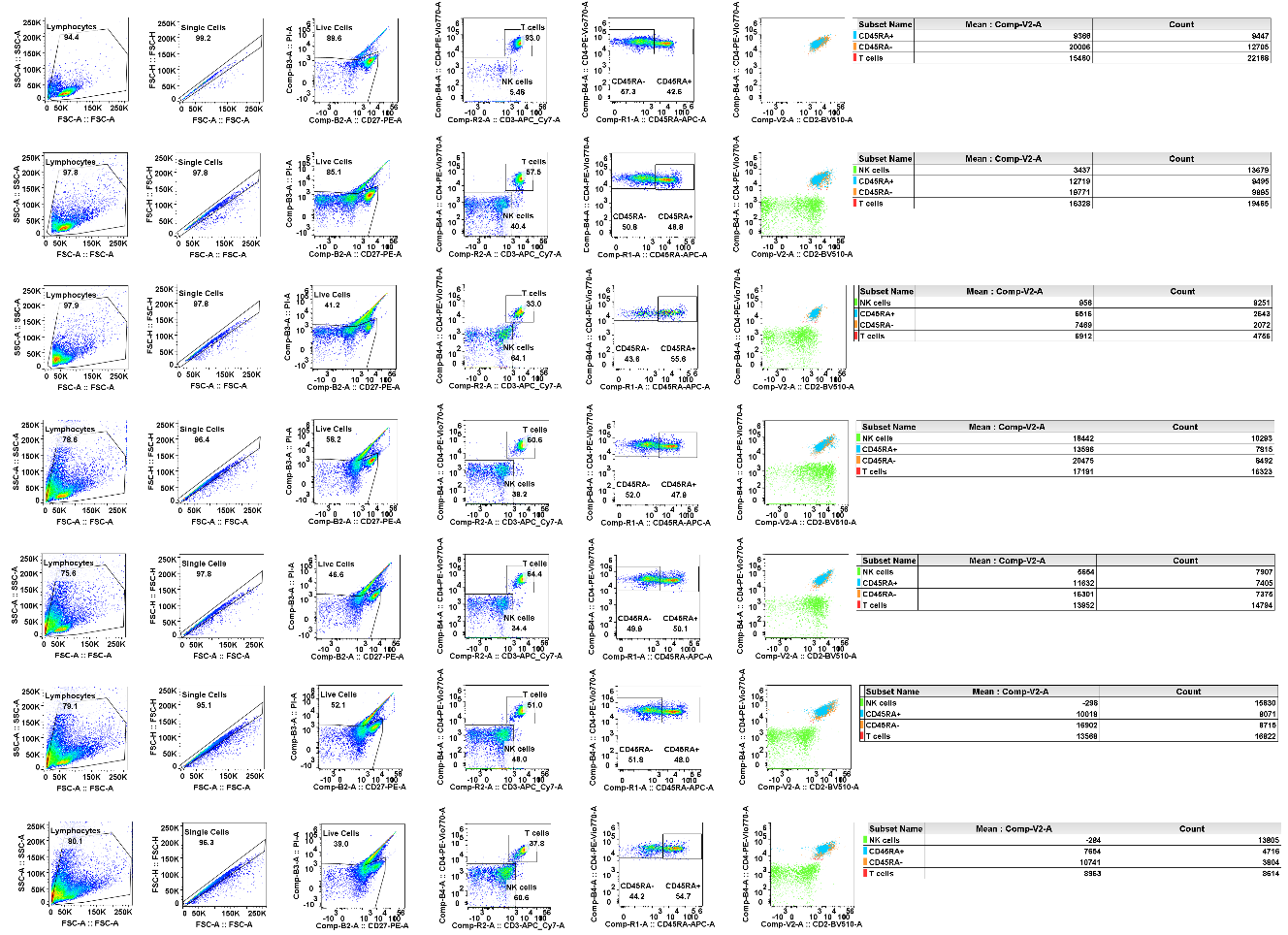


**CD4^+^ T cells**

**no treatment**

**Day 0**

**IgG1**

**alefacept**

**Day 21**

**IgG1**

**IgG1**

**alefacept**

**alefacept**

**+ BD FastImmune^TM^ α-CD2**

**CD2-MFI**

**Figure S3. Gating strategy for primary and expanded NK cell and auto/allo CD4^+^ T cell killing assay.** CD4^+^ T cells with no treatment, day 0 (primary) NK cells and day 21 (expanded) NK cells and self (auto) CD4^+^ T cells with 10 µg/mL alefacept or IgG1 control antibody after co-culture for 19 hours at E:T = 1:1.

**10**

**0.001**

**10**

**0.001**

**A**

**μg/mL**

**B**


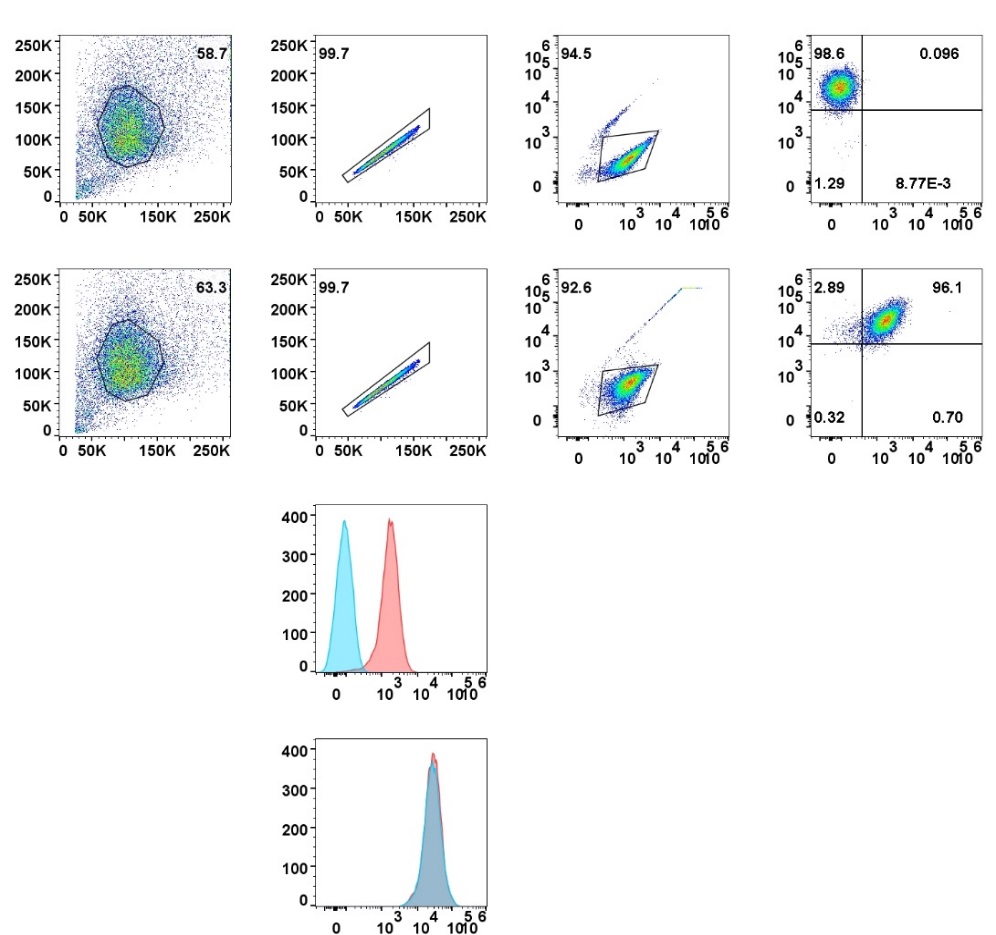


**unstained**

**CD16.NK-92**

**CD16-APCVio770**

**CD16**

**GFP**


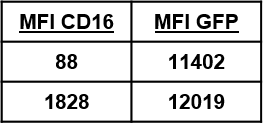


**SSC-A**

**FSC-H**

**PI viability**

**GFP**

**FSC-A**

**FSC-A**

**B2 channel**

**CD16**

**Figure S4. Alefacept dose response and CD16.NK-92 staining.** A) CD16.NK-92 and CD4^+^ T cells from healthy donor were co-cultured at E:T = 1:1 and 0.1:1 for 19 hours with serial dilutions of alefacept. Percent dead CD4^+^ T cells was determined by flow cytometry viability dye staining. B) Surface staining and flow cytometry of CD16.NK-92 with α-human CD16.

**A**

**B**

**Figure S5. 10 gray irradiated CD16.NK-92 target CD45RA- memory CD4+ T cells and this is enhanced with alefacept.** CD4^+^ T cells and 10 gray irradiated CD16.NK-92 were co-cultured at E:T= 0.5:1 for 19 hours (n = 4 healthy donors) with no treatment, CD16.NK-92 and 10 µg/mL alefacept or IgG1 control antibody. A) CD2 MFI of each cell subset in killing assay. B) Percent cytotoxicity of each cell subset in killing assay.


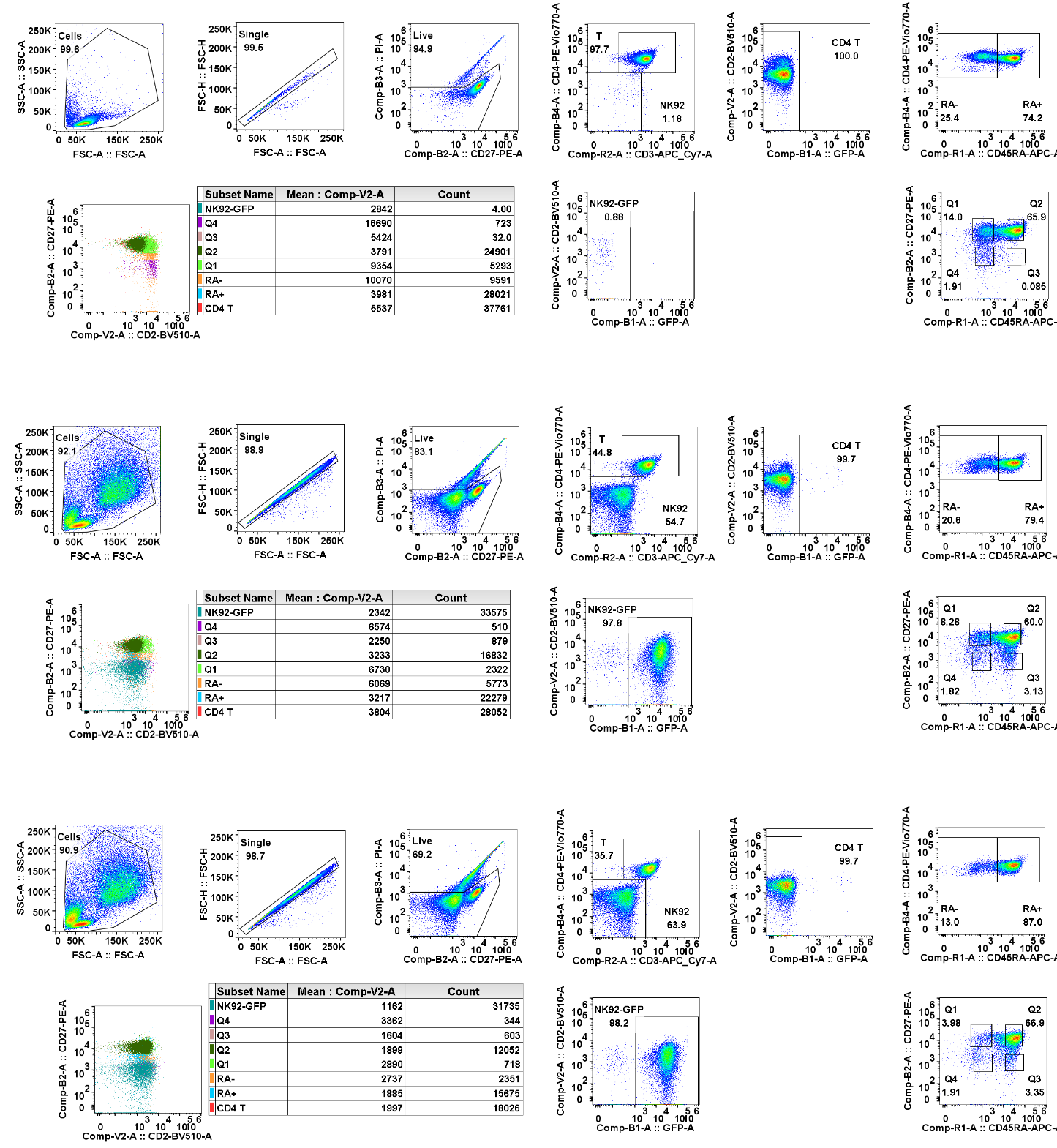


**Healthy donor F26**

**CD4^+^ T cells**

**no treatment**

**+ CD16.NK-92**

**E:T = 0.5:1**

**10 µg/mL IgG1**

**+ CD16.NK-92**

**E:T = 0.5:1**

**10 µg/mL alefacept**

**CD2-MFI**

**CD2-MFI**

**CD2-MFI**

**Figure S6. Gating strategy for CD16.NK-92 and CD4^+^ T cell target killing assays (healthy donor and HIV^+^).** CD4^+^ T cells and CD16.NK-92 were co-cultured at E:T= 0.5:1 for 19 hours (healthy donors) or 19 hours and 3 days (HIV+) with no treatment, CD16.NK-92 and 10 µg/mL alefacept or IgG1 control antibody.

********

**B**

**A**

**C**

**Figure S7. NK92-CD16V-GFP and alefacept reduce HIV DNA after 3 day co-culture**.

A) Table of qPCR HIV gag Ct values in triplicate with HIV^+^ CD4^+^ T cells alone, CD16.NK-92 and 10 µg/mL alefacept or IgG1 control antibody. B) HIV gag standard curve with ACH2 (harbors 1 stably integrated copy of HIV per cell) is serially diluted into parental cell line A3.01 (HIV-) down to 1 copy HIV/well in which 1 copy of HIV *gag* is readily detectable by qPCR. C) HIV DNA copies/well as determined by StepOnePlus and RPP30. Dotted line represents the limit of detection (L.O.D.) of 1 copy of HIV *gag* DNA copy/well.
